# Sanitary Science in the Camp—Being the Conclusions of the Commission Appointed by the British Government to Inquire into the Sanitary Condition of the Army in the Crimea

**Published:** 1861-09

**Authors:** George W. Wilde

**Affiliations:** London


### SANITARY SCIENCE IN THE CAMP.

*Being the Conclusions of the Commission appointed by the British Government, to Inquire into the Sanitary Condition of the Army in the Crimea.*

BY GEORGE W. WILDE, M. D., of London.

Although it has been my fortune to spend a considerable portion of my professional life abroad, I still take a deep interest in the political affairs of my native country, and would gladly add whatever of influence or power I may possess to sustain a Government, the wisest and most beneficient ever created, in its efforts to maintain its supremacy. That the Federal Government will have an army of any dimensions which it may require, I do not doubt; nor do I doubt the results of the war. The old flag—the stars and stripes—will long continue to float an emblem of a united and a free government; not only over every inch of our national domain, but in every harbor of the civilized world.

But the question of vital interest to me is, will there not be an immense and needless sacrifice of life from preventible diseases in an army so quickly collected from among the people, and so unaccustomed to the habits and pursuits of the soldier's life? I am glad to learn that this subject has already engaged your attention, and that measures have been taken by government to remedy these evils.

My attention has been repeatedly called to the sanitary condition of troops in my travels on the continent and in the East; and my observations have given me the liveliest apprehensions in regard to the health of the vast army now assembled along the borders of the Southern States. Unless the highest degree of intelligence in matters relating to the management of troops in camp, and in the field, is possessed by the medical staff, the disasters by sickness are far more to be apprehended than reverses on the battle-field; as American physicians have had little actual experience in military life, and as the medical literature of military surgery has to be obtained, for the most part, from abroad.

Yet, it has occurred to me, that there is a large fund of facts relating to the hygiene of armies contained in public documents, and hence quite inaccessible—at least to the majority of medical men. The record of many of these investigations are rich in the fruits of sanitary science applied to armies. I believe I cannot do my professional brethren at home, who are now engaged in their country's service, a better service than by giving at some length, and in detail, except where condensation is possible, the results of some of these official inquiries.

The following are the conclusions of the Sanitary Commission sent to the Crimea by the British government, to inquire into the sanitary condition of the troops. They will be seen to embrace the most important facts relating to the hygiene of camps, and are the result of a series of long and laborious observations:

PRACTICAL CONCLUSIONS RESPECTING THE CAMP.

I. That by far the greater part of the disease and mortality existing in the camp, when the Commission arrived in the Crimea, was due to zymotic maladies, such as cholera, fever, diarrhœa, and dysentery.

That besides the effects of topographical and climatic peculiarities connected with the occupation, and making allowance for the predisposing influence of other conditions, to which the troops had been exposed, the prevalence of zymotic maladies was obviously connected with local favoring causes, essentially the same in kind as those observed in civil life, especially in rural districts, namely:

Damp.

Impure Air.

(Although in a minor degree) Impure Water.

II. Attacks of zymotic disease were observed to be connected with the three following sources of dampness:

A wet subsoil; a retentive surface soil; confined locality.

1. Of these three conditions, *a wet subsoil* occasioned the largest proportional amount of sickness.

The experience of the 79th Regiment, and that of the 31st and Royal Artillery, who were successively camped on the same ground, below Marine Heights, proves that one of the worst sites for a camp is that in which a thin bed of porous material rests upon an impervious bed beneath, which retains the water, and keeps the subsoil charged with it, while the surface may afford little or no indication of the fact.

Dangerous sites of this kind were often marked by a greener or more vigorous vegetation than that of the surrounding district, or by water-springs coming to the surface, or by evening fogs setting over them sooner than over the adjacent country.

Before selecting positions for camps in unknown ground, it would be very advisable to dig trial holes a few feet deep, to ascertain what is the condition of the subsoil drainage, and not risk the health of the men in camping on ground in which these trial holes show the presence of water near the surface.

Should it be necessary, for military reasons, to hold a position on a wet subsoil, the whole should, if practicable, be thoroughly drained by deep trenches, and if there be a hill-side or water-shed above the ground, the surface water from it should be turned aside from the site by deep, catch-water drains, as was done with the camp of the Highland Division at Kamara.

If the position be such that deep trenching and draining cannot be carried out, it is in the highest degree probable that if held for any length of time, it will be at a considerable sacrifice of the force.

2. *The retentive character of clay surface soils*, and the difficulty of draining such soils, render it advisable to avoid them as camping-grounds, when it is possible to do so.

Wet clay soils keep the air near the ground damp and cold, and they affect the atmosphere of tents and huts in a similar manner. There was sufficient proof of their injurious effects on the health of troops in the Crimea.

Where such soils must be occupied, for military reasons, the defects in the natural drainage should be remedied, as far as practicable, by trenching the ground, and by trenching the site of every hut and tent separately, connecting the hut and tent drains with the larger trenches. In this way, not only are the sites and the vicinity of the huts and tents kept comparatively dry, but the surface water is more readily removed, the exhalations from the damp soil diminished, and the air purified. The experience of the army in the Crimea showed the very beneficial effects of this surface drainage and trenching on the health of the troops.

3. *Dampness of the air, arising from the nature of the locality*, proceeds from the topographical peculiarities of the ground preventing a free circulation of the air, and the atmosphere becoming stagnant, and charged with moisture and emanations from the ground. The valley of Karani above Kadikoi afforded an illustration of this, in certain states of the weather.

It was observed in other parts of the seat of the war in the East, that damp white mists, settling in valley or hollows occupied by troops, had been the precursors of epidemic diseases, especially of cholera. All valleys are at times exposed to similar occurrences, especially such as contain stagnant lakes. An unhealthy and stagnant state of the air is sometimes increased by brushwood or trees.

There is often no escape from epidemic sickness occuring among troops from the occupation of such positions; they should, therefore, be avoided or abandoned.

III. The evils resultiug from these local causes of dampness were not unfrequently aggravated by the manner of pitching tents and erecting huts. Want of due preparation of the ground and defective drainage of the site, often led to a damp state of the air within huts and tents, and induced a tendency to fevers.

Deep trenching round the tent-site, as already mentioned, is the best remedy, and in case of huts, the site should be isolated from the surrounding ground, and the area to be occupied by the hut, drained by a trench dug round it at least a foot below the level of the floor.

If it be not practicable to drain the subsoil, and if the position must be held, adequate provision should be made with any materials at hand for raising the beds of the men above the ground.

Huts should never be banked up with earth against the wood. The experience in the Crimea has shown that it is a dangerous practice, for it used to be a common cause of fevers. An interior lining, even of old newspaper, affords a much better, and at the same time a perfectly safe protection from draughts.

The flooring of huts should be occasionally raised, the surface of the ground below cleansed, and quicklime and charcoal strewed over it.

For hospital huts, an interior lining of boards, or building a rough rubble stone wall outside, as was done in many of the regimental hospitals, affords the requisite protection from weather, and from sun heat.

IV. The camp before Sebastopol was, generally, remarkably clean when first visited; but there were in certain situations sources of atmospheric impurity from putrescent organic effluvia, likely to influence injuriously the health of the troops. The chief of these were:

Picketting-grounds, and manure heaps.

One or two slaughtering-places, and latterly the large cattle depot and slaughtering-place at Kadikoi.

The graveyards and putrid marsh near Balaklava.

Latrines kept too long open, and exposing too large a surface.

When an army can shift its ground at will, danger to health from similar evils can always be avoided by doing so.

When, on the other hand, an army is tied to its position for a length of time, the camp becomes a town, and is subject to all the sanitary defects of towns, as these existed before the introduction of the first great step that was taken for improving the public health, namely, the introduction of paving.

Picketting of horses saturates the ground they occupy with organic matter. In like manner, accumulations of manure, if allowed to remain, saturate the ground they cover. Filth of any kind is washed into the ground by the rains, or trodden into by the steps of men and animals, and must necessarily give off impure emanations under the joint action of sun, heat and moisture.

To avoid the injurious consequences likely to arise from these circumstances, it is indispensably necessary to observe the most scrupulous cleanliness over the whole surface and vicinity of a camp. All refuse should be at once swept up, and removed to a distance. None should ever be allowed to accumulate within, or in the immediate vicinity of a camp.

Bones and refuse of food can be most easily disposed of by burial.

Stable litter and all inflammable refuse should be carefully burned. The usual method of forming heaps of litter and firing it, is imperfect. Before being fired, it should always be opened up, to admit the air to dry it, and to expedite the combustion. Manure heaps burn with difficulty, if left on the ground for any length of time before they are fired.

Carcases of animals and offal should be buried to a sufficient depth below the surface. Three feet is enough under ordinary circumstances. Refuse charcoal dust thrown over tainted ground will assist in deodorizing it, or, if that be not obtainable, the burning of stable litter on the spot will furnish sufficient charcoal for the purpose.

Latrines should be made narrow and deep; a quantity of earth should be thrown into them each day, until they are filled within two feet of the surface, after which the latrine should be filled up, and another dug.

When an army requires to occupy the same surface of ground for years, it would be unsafe to bury the refuse in the ground, because eventually the soil would become saturated with organic matter, and dangerous to health.

In such a case, the construction of furnaces to consume every organic product of the camp, is far the best and safest proceeding. Speedy collection, removal, and destruction by fire of all such refuse matters obviates any risk of danger from them.

V. *Atmospheric impurities arisi[illegible]rom overcrowding and defective ventilation of tents and [illegible]*, were a frequent predisposing cause of zymotic disease.

Were it practicable in warfare to diminish materially the number of men sleeping in tents, it would be advisable to do so.

But considering the limited transport at the command of an army in the field, the injurious consequences of overcrowding may, to a considerable extent, be obviated by a free ventilation of huts, and by improving the construction of tents and marquees, by introducing effectual means of ventilation round the top of the poles.

In the case of huts, ridge ventilation is the most efficient.

Lime-washing huts inside, especially hospital huts, purifies the air; lime-washing of huts outside protects them, to a certain extent, from the intense sun's rays, and keeps them cooler within.

The usual effects of striking tents and shifting ground is an excellent means of avoiding the effects of saturation of the earth by emanations proceeding from the breath and bodies of the men.

VI. The condition in which the water was drawn for use in the camp was likely, especially during the prevalence of cholera, to aggravate the severity of the disease, although not to a great degree.

It is always desirable that water for drinking and cooking purposes should be as nearly as possible destitute of color, taste, or smell. Anything that interferes with these three natural tests is more or less injurious to health; but marsh water, however apparently pure, is not wholesome.

All engineering works for supplying camps with water should comprehend—

The selection of the purest obtainable source.

The delivering the water for use as pure as it is at its source.

If it be necessary to pound the water, the tanks should be covered.

Water should, if practicable at all, never be drawn by dipping, if it be rendered muddy in the act of being so drawn.

If a source of water of sufficient purity be not obtainable, the water should be filtered. A filter may be made with sorted gravel, clean sand, and charcoal.

Every trough for supplying horses should have a separate inlet and overflow.

### GENERAL CONCLUS[illegible]S FROM THE WHOLE EXPERIENCE.

I. That as scurvy, a[illegible] the forms of disease connected with it, almost disappeared from the army under the influence of improved diet, clothing, etc., so, in like manner, zymotic diseases, the destructive effects of which mainly depend on breathing a humid, tainted atmosphere, declined on the carrying out of suitable sanitary works and measures.

II. That men just arrived in a new country are especially liable to suffer from prevailing zymotic maladies. That any given number of reinforcements will not compensate to the service for the loss of the same number of the original force from these diseases, and hence the necessity for effective sanitary precautions is doubly imperative, whether as regards the abatement of local favoring conditions, or the discovery and immediate treatment of the premonitory stages.

III. As the result of their whole experience, the Commissioners beg to express their opinion, that, inasmuch as the neglect of military hygiene, whether as regards the soldier personally, or the sanitary condition of camps, barracks, and hospitals, has hitherto, in all countries, climates, and seasons, been the cause of the largest amount of loss in armies, the whole subject, closely connected as it is with the physical efficiency of Her Majesty's forces, demands in future a practical development commensurate with its importance to the public service.—*Am. Med. Times.*

